# Effects of evidence-based clinical practice guidelines for breast cancer in health care quality improvements. A second systematic review.

**DOI:** 10.12688/f1000research.126126.1

**Published:** 2022-10-25

**Authors:** Anggie Ramírez-Morera, Mario Tristán, Jordan Salazar-Vargas, Ana Leonor Rivera-Chavarría

**Affiliations:** 1 Cochrane Central America & Caribbean Spanish, IHCAI Foundation, San José, San José, 10101, Costa Rica; 2 Universitat Autònoma de Barcelona, Barcelona, Catalunya, 08041, Spain; 3 Caja Costarricense de Seguro Social, San José, San José, 10105, Costa Rica; 4 Instituto Costarricense de Investigación y Enseñanza en Nutrición y Salud, Tres Ríos, Cartago, 42250, Costa Rica

**Keywords:** Clinical Practice Guidelines; CPG; effect; health care quality.

## Abstract

**Background:** Traditionally, EB-CPGs have been believed to mainly improve the quality and consistency of health care, but this claim must be conclusively proven. We used the Donabedian three-dimensional model (structure, process, and patient outcomes) to assess improvements in the quality of medical care derived from implementing EB-CPGs. This study corresponds to the second systematic review carried out as a series of studies on different clinical issues that aim to evaluate the effectiveness of the application of the EB-CPG for improving the quality of care.

**Methods:** We followed the methods described by the Cochrane Handbook and presented a descriptive analysis because of the high heterogeneity found across the included studies. We searched the Cochrane Central Register of Controlled Trials, PubMed, and EBSCO Host databases, as well as the grey literature, between 1990 and April 2021. No language restrictions were applied. Only randomised clinical trials (RCTs) were selected.

**Results:** Of the total of 364 interventions included in the eleven RCTs evaluated, 11 (3%) were related to healthcare structure, 51 (14%) to the healthcare delivery process and 302 (83%) to patient outcomes. Regarding the impact of using the EB-CPGs, in 303 interventions (83%), there were no significant differences between the control and experimental groups. In 4 interventions (1%), the result favoured the control and intervention groups in 57 of the interventions (16%).

**Conclusions:** Our study showed that EB-CPGs slightly enhanced the quality of health care in the three dimensions described by Donabedian. Future RCTs should improve their design and methodological rigour by considering the certainty of the evidence supporting the EB-CPGs recommendations. In that context, broader analyses could be performed, having more concise hypotheses for further research.

Registration: PROSPERO CRD42020205594

## Introduction

The emergence of the Evidence-Based Clinical Practice Guidelines (EB-CPGs) in the 1990s has improved decision-making for healthcare personnel and patients with different health conditions ( IOM, 1990; Weisz *et al.*, 2007).

Worldwide, significant efforts have been made to develop and implement EB- CPGs based on scientific evidence. The term “evidence-based” means that the recommendations described in the CPGs derive from the best scientific findings and the highest quality of evidence obtained from applying unbiased, transparent, and rigorous methods to support clinical care ( Watters, 2008; Linskey, 2010).

EB-CPGs are statements that include recommendations intended to improve the quality and consistency of health care ( Woolf *et al.*, 1999; Kwan, 2004) and to assist clinical staff and patients in the decision-making process ( Alonso-Coello *et al.*, 2010; IOM, 2011).

This study corresponds to the second systematic review of a series of studies on different clinical subjects, which aim to assess the success of the use of EB-CPGs ( Ramírez-Morera *et al.*, 2019). Therefore, we evaluated the effectiveness of the application of EB-CPGs for the improvement of the quality of health care in three dimensions: structure, process, and patient outcome in the management of breast cancer (Donabedian Model, Donabedian, 1988).

Studies measuring the effects of EB-CPGs on the quality of health care have mainly focused on the effects on clinical practice ( Lugtenberg *et al.*, 2009), state that some international reviews have demonstrated that most of the studies have resulted in significant improvements to the process of care. However, few studies have focused on the effects of measures on patients’ health outcomes.

We considered for this review breast cancer disease because this is the most common malignancy in women around the world ( Ghoncheh *et al.*, 2016). Although survival has improved in the last 30 years mainly because of the implementation of early detection programs, it still registers 2.3 million new diagnostics in women during 2020 and 685 000 deaths within the same year ( WHO, 2020).

Strategies to control and prevent this type of cancer must be a high priority for health policymakers ( Ghoncheh *et al.*, 2016). According to the above, hundreds of breast cancer guidelines have been published worldwide to reduce its negative impact on men’s and especially on women’s health.

This review is relevant because there is a growing number of EB-CPGs in different essential areas, and their actual impact on relevant outcomes needs to be assessed. Few systematic reviews evaluate the effect of EBGPC in improving health care; these focus solely on one clinical entity and cover a country or region ( Grimshaw *et al.*, 1993; Worrall *et al.*, 1997; Lugtenberg *et al.*, 2009; Ricci-Cabello *et al.*, 2020). For this reason, we reviewed the evidence on the benefits of implementing EB-CPG to improve the quality of care. This review responds to the need for EB-CPG research synthesis on the overall quality of health care delivery.

This systematic review contributes to meeting the need for research synthesis about EB-CPG by assessing the overall quality of health care delivery. Besides, we visualise the need for a systematic review that conclusively demonstrates the pragmatic impact that evidence-based recommendations have on breast cancer patients.

## Methods

We conducted a systematic review to identify and analyse the effect of EB-CPGs on health care quality improvement within the Donabedian Model dimensions: structure, process, and results ( Donabedian,1988). We followed the Cochrane Handbook methodological recommendations described by Higgins *et al.* (2022a).

This review is registered at PROSPERO (ID: CRD42020205594).

### Study search

The research question was translated into the PICO framework for guiding the study search and the criteria selection ( Table 1). We developed a method to incorporate the methodological component of the search strategy combined with selected index and free-text terms.

**Table 1.** Structure of the clinical question.

Problem	Population	Intervention	Comparison	Outcome
Effects of evidence-based clinical practice guidelines for breast cancer in health care quality improvements	Healthcare professionals involved in breast cancer care	EB-CPGs for the management of breast cancer	Standard care for breast cancer	The impact of EB-CPGs for breast cancer on improving the quality of health care (structure, process, patient outcomes)

We explored the following electronic databases for primary studies: Cochrane Central Register of Controlled Trials (CENTRAL), Cochrane Library, including the Cochrane Effective Practice and Organisation of Care (EPOC) group specialised register, Pubmed, Scopus, EBSCO, Academic Search Complete, CINAHL, Biomedical Reference Collection: Comprehensive, APA PsycInfo, Nursing & Allied Health Collection: Comprehensive, Alt HealthWatch, SPORTDiscus with Full Text, Psychology and Behavioral Sciences Collection, Health Source: Nursing/Academic Edition, Biomedical Reference Collection: Basic, AMED - The Allied and Complementary Medicine Database, Consumer Health Complete, Cochrane Database of Systematic Reviews, Cochrane Methodology Register, Rehabilitation & Sports Medicine Source, AgeLine, Global Health, International Pharmaceutical Abstracts, MasterFILE Premier, Rehabilitation & Sports Medicine Source, LILACS, and Health Technology Assessment Database. We also searched the Science Citation Index and Social Sciences Citation Index for papers that refer to studies included in the review.

The PubMed search strategy was executed in the other databases using the appropriate controlled vocabulary. Searching for other resources included grey literature from different sources and hand searching of those high-yield journals and conference proceedings that have not already been hand searched on behalf of the Cochrane Collaboration.

Authors of relevant papers were contacted regarding any further published or unpublished work. Authors of other reviews in the field of effective professional practice were contacted regarding relevant studies of which they may be aware. We searched for studies published between January 1990 and April 2021. The search strategy was not restricted by language. An advanced search strategy and results are available as extended data, Appendix 1 ( Ramírez-Morera *et al.*, 2022).

For the management of bibliographic references of the articles found, the web application “Sciwheel Reference Manager & Generator” was used ( Sciwheel, 2022).

### Studies selection

The studies found through the search strategy were screened by two reviewers (AR, JS), and discrepancies about study selection were resolved by a third reviewer (MT). Inclusion criteria were: 1. Randomised Clinical Trial (RCT) or cluster-type RCT measuring the impact of using any implementation model versus passive dissemination or no use of the EB-CPG. 2. The studies evaluated the impact on any of the three domains described in the Donabedian model (structure, process, and patient outcomes) for using EB-GPC in treating breast cancer. 3. No language restriction. 4. Published studies from 1990 to 2021.

### Data extraction

Three authors (AR, JS, ALR) independently undertook data extraction. They used a modified version of the Cochrane Collaboration EPOC Group “Data Collection Checklist”, employing an electronic datasheet ( EPOC, 2019).

To assess the bias risk, we used standard Cochrane methods described in chapters 8, 10 and 23 of the Cochrane Handbook for Systematic Reviews of Interventions ( Higgins *et al.*, 2022b; Sterne *et al.*, 2019). In the case of RCTs, bias resulting from several types of systematic errors was assessed according to methods described by Cochrane ( Higgins *et al.*, 2022c) and following the RoB2 instrument ( Sterne *et al.*, 2019).

All studies deemed eligible for the review were assessed independently by the review authors (AR, MT), and discrepancies were resolved by discussion. A summary of the risk of bias assessment is presented as part of the characteristics of included studies table.

We did not find cluster-type RCTs; then, we did not apply what is stated in chapter 23 of the Cochrane Handbook for Systematic Reviews of Interventions ( Higgins *et al.*, 2022d) about assessing cluster RCT following the RoB2 instrument ( Sterne *et al.*, 2019).

An analysis of the quality of the evidence related to each of the outcomes was performed using the GRADE approach ( Schünemann *et al.*, 2013). We assessed the certainty of the body of evidence for each key outcome as “high”, “moderate”, “low”, or “very low”, using the GRADEpro GDT platform ( GRADEpro, 2021).

Data were analysed using Review Manager software, version 5.4 ( RevMan, 2020). Revman default templates for data extraction were modified to show the results in a simplified way.

We found very high variability between the measures of effect within the included studies in this review. Then, we decided not to perform a meta-analysis and, therefore, neither to measure statistical heterogeneity.

## Results

### Study identification and selection

**Figure 1.**
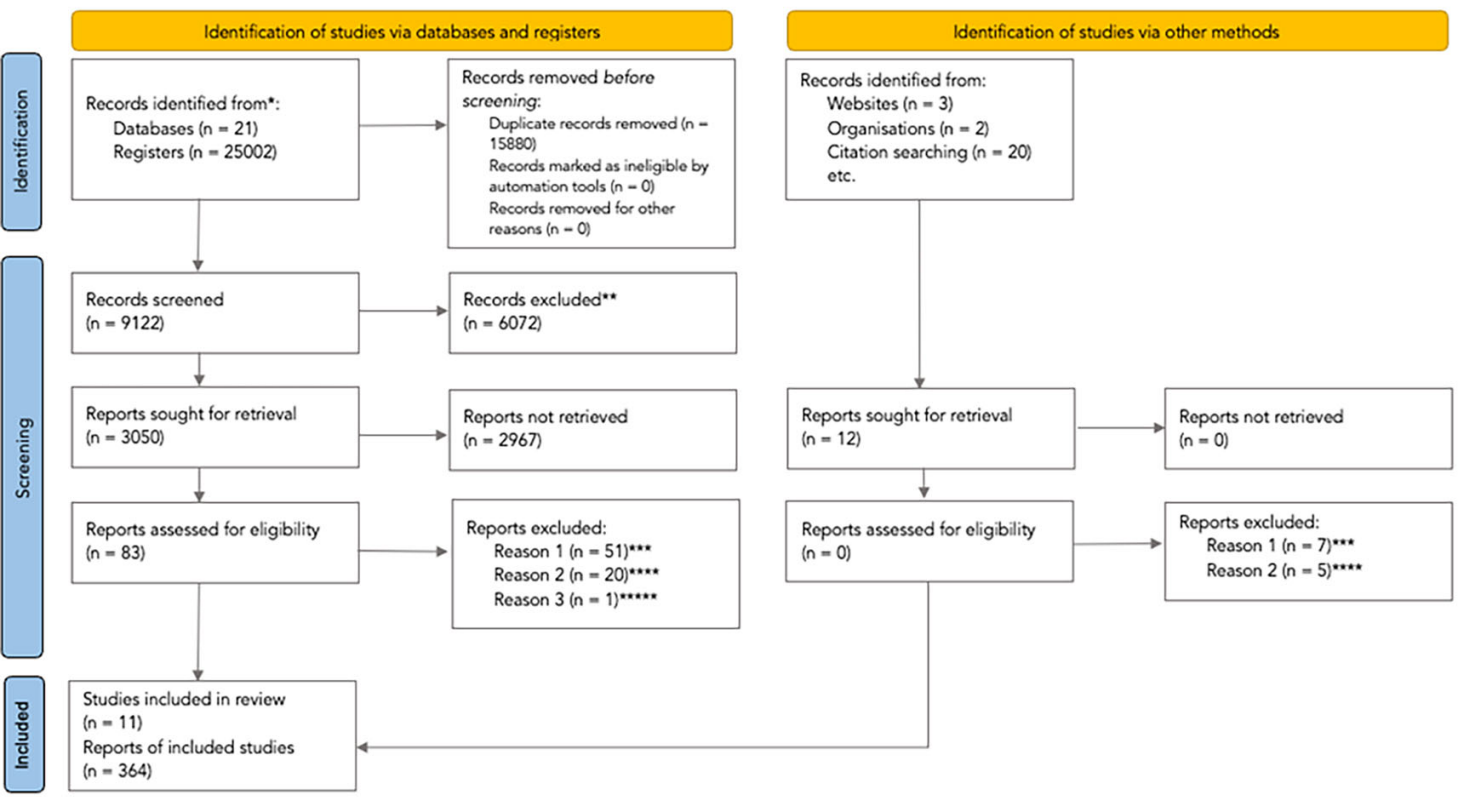
PRISMA flowchart of the studies selection process (PRISMA **;** Page *et al.*, 2021). * The appendix 1 shows the number of records identified from each database or register searched. ** All records were excluded by the authors. Excluded after screening by Title/Abstract. *** The records were not randomised controlled trials. **** The records did not evaluate clinical practice guidelines. ***** They published the results included by Irene *et al.* (2019), whose study described a broader methodology and reported more detailed results. From: Page MJ, McKenzie JE, Bossuyt PM, Boutron I, Hoffmann TC, Mulrow CD, *et al.* The PRISMA 2020 statement: an updated guideline for reporting systematic reviews. BMJ 2021;372:n71. doi: 10.1136/bmj.n71. For more information, visit: http://www.prisma-statement.org/

The process describing the analysis of studies retrieved through the systematic search is content in the PRISMA flowchart ( Figure 1, Page *et al.*, 2021). We retrieved 25002 studies from database searching and 20 studies were found from additional sources identified. We excluded 15900 duplicated records, and 6072 studies were excluded after screening by title and abstract. We assessed 83 articles at the full-text level, excluding 72 references which did not meet the selection criteria: 51 (71%) were not randomised controlled trials, and 20 (28%) did not evaluate clinical practice guidelines.

Stark *et al.* (2018) was excluded from this systematic review. They published the results included by Irene *et al.* (2019), whose study described a broader methodology and more detailed reported results. The list of excluded studies and reasons for exclusion are available as extended data, Appendix 2 ( Ramírez-Morera *et al.*, 2022).

### Characteristics of the included studies

We included 11 RCTs analysing CPGs for breast cancer ( Boekhout *et al.*, 2015; Greenlee *et al.*, 2016; Grunfeld *et al.*, 2011; Hershman *et al.*, 2013; Irene *et al.*, 2019; Klinkhammer-Schalke *et al.*, 2012; Knobf *et al.*, 2016; Maly *et al.*, 2017; Park *et al.*, 2015; Park *et al.*, 2019; Smith-Turchyn *et al.*, 2020). The list of studies selected after screening and assessing the full text is available as extended data, Appendix 3; Ramírez-Morera *et al.*, 2022).

The trials were published between 2011 and 2020, most from 2014 to 2016 (5, 46%). Approximately 55% (6) were carried out in the United States of America. The clinical practice guidelines examined within the selected trials were follow-up (11, 100%) and treatment (4, 36%). When evaluating the outcome categories involving the clinical practice guidelines quoted, most referred to the quality of life (7, 63%). A summary of the characteristics of the included studies (n=11) is available ( Table 2). A broader description of these characteristics is available as extended data ( Appendix 4; Ramírez-Morera *et al.*, 2022) and the list of the CPGs examined in the included studies ( Appendix 5; Ramírez-Morera *et al.*, 2022).

**Table 2.** Characteristics of all included studies (n=11).

Study characteristics	n (%)	Citation
Country		
Canada	3 (27%)	Boekhout *et al.*, 2015; Grunfeld *et al.*, 2011; Smith-Turchyn *et al.*, 2020.
Germany	1 (9%)	Klinkhammer-Schalke *et al.*, 2012.
South Korea	1 (9%)	Park *et al.*, 2015.
USA	6 (55%)	Greenlee *et al.*, 2016; Hershman *et al.*, 2013; Irene *et al.*, 2019; Knobf *et al.*, 2016; Maly *et al.*, 2017; Park *et al.*, 2019.
Publication year		
2011-2013	2 (18%)	Grunfeld *et al.*, 2011; Klinkhammer-Schalke *et al.*, 2012.
2014-2016	5 (46%)	Boekhout *et al.*, 2015; Greenlee *et al.*, 2016; Hershman *et al.*, 2013; Knobf *et al.*, 2016; Park *et al.*, 2015.
2017-2020	4 (36%)	Maly *et al.*, 2017; Irene *et al.*, 2019; Park *et al.*, 2019; Smith-Turchyn *et al.*, 2020.
Guideline scope *		
Treatment	4 (36%)	Klinkhammer-Schalke *et al.*, 2012; Knobf *et al.*, 2016; Maly *et al.*, 2017; Park *et al.*, 2019.
Follow up	11 (100%)	Boekhout *et al.*, 2015; Greenlee *et al.*, 2016; Grunfeld *et al.*, 2011; Hershman *et al.*, 2013; Irene *et al.*, 2019; Klinkhammer-Schalke *et al.*, 2012.; Knobf *et al.*, 2016; Maly *et al.*, 2017; Park *et al.*, 2015; Park *et al.*, 2019; Smith-Turchyn *et al.*, 2020.
Outcome category *		
Exercise	4 (36%)	Boekhout *et al.*, 2015; Knobf *et al.*, 2016; Park *et al.*, 2019; Smith-Turchyn *et al.*, 2020.
Nutrition	3 (27%)	Greenlee *et al.*, 2016; Knobf *et al.*, 2016; Park *et al.*, 2019.
Quality of life	7 (63%)	Grunfeld *et al.*, 2011; Hershman *et al.*, 2013; Irene *et al.*, 2019; Klinkhammer-Schalke *et al.*, 2012.; Maly *et al.*, 2017; Park *et al.*, 2015; Smith-Turchyn *et al.*, 2020.
Treatment	3 (27%)	Boekhout *et al.*, 2015; Klinkhammer-Schalke *et al.*, 2012; Knobf *et al.*, 2016.

* Percentages exceed 100% because the categories are not mutually exclusive (i.e., some studies involved more than one type of guideline scope and more than one outcome category).

Two different studies ( Maly *et al.*, 2017; Irene *et al.*, 2019) evaluated the CPG for Fertility preservation for patients with cancer: American Society of Clinical Oncology clinical practice guideline update ( Loren *et al.*, 2013). Park *et al.* (2015) and Smith-Turchyn *et al.* (2020) analysed the American College of Sports Medicine roundtable on exercise guidelines for cancer survivors ( Schmitz *et al.*, 2010).

Additionally, Maly *et al.* (2017) examined the latest version of the CPG by Khatcheressian *et al.* (2013), American Society of Clinical Oncology 2006 update of the breast cancer follow-up and management guidelines in the adjuvant setting, previously analysed by Hershman *et al.* (2013) and Greenlee *et al.* (2016). They assessed Khatcheressian *et al.* (2006).

Grunfeld *et al.* (2011) reported the results until 12 months. Extended results (up to 24 months) for the same study were published by Boekhout *et al.* (2015). Hershman *et al.* (2013) and Greenlee *et al.* (2016) published different results of the same study due to the questionaries or scale utilised for measuring outcomes. The first was more interested in the quality of life and treatment satisfaction, and the second in lifestyle behaviours. We found that Knobf *et al.* (2016) and Park *et al.* (2019) published the same study. Knobf *et al.* (2016) focused on describing the effect of exercise on bone density, while Park *et al.* (2019) described the results concerning the quality of life.

*Risk of bias assessment*

We assessed the risk of bias with the RoB2 instrument in the eleven included RCTs following the methods described.

We found that a low risk of bias prevailed (6; 55%) in domain 1: randomisation process. Some concerns occurred in domain 2: deviations from the intended interventions (7; 64%). A low risk of bias was found for domain 3: missing outcome data (10; 91%) and domain 4: measurement of the outcome (8; 73%). Finally, we found some concerns in domain 5: selecting the reported result (7; 64%).

**Figure 2.**
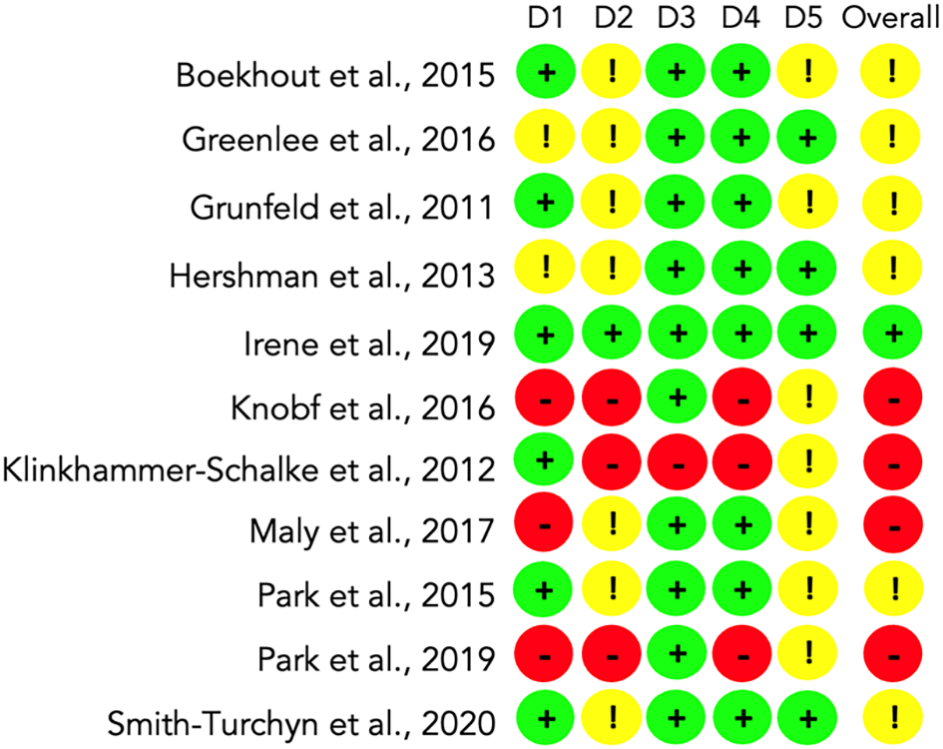
Analysis of the Risk of Bias (RoB2) for all included studies (n=11). D1: Randomisation process. D2: Deviations from the intended interventions. D3: Missing outcome data. D4: Measurement of the outcome. D5: Selection of the reported result. Overall risk of bias.

Overall, one study (9%) reported low risk ( Irene *et al.*, 2019), some concerns arise from 6 (55%) studies ( Grunfeld *et al.*, 2011; Hershman *et al.*, 2013; Boekhout *et al.*, 2015; Park *et al.*, 2015; Greenlee *et al.*, 2016; Smith-Turchyn *et al.*, 2020) and high risk occurred in 4 (36%) studies ( Knobf *et al.*, 2016; Klinkhammer-Schalke *et al.*, 2012; Maly *et al.*, 2017; Park *et al.*, 2019). A summary of the results is available in Figure 2.

Most of the risk of bias found in the included studies occurred due to the lack of existence or some level of blinding. In some cases, outcome data were not available entirely for all randomised participants. Four studies did not analyse the intention to treat, and some outcome measurement methods were not adequately described. A broader description of the risk of bias assessment is available as extended data in Appendix 6 ( Ramírez-Morera *et al.*, 2022).

### Quality of evidence assessment

The studies showing the lowest risk of bias ( Irene *et al.*, 2019) and one resulting in the highest risk of bias ( Park *et al.*, 2019) were chosen to be evaluated with the GRADE methodology and to build a summary of the findings. We decided to grade only four outcomes per study described, including two reporting statistically significant results and two not statistically significant results. Performing a GRADE table provided the rank of possible grades for the certainty of the evidence found in the 362 interventions from the 11 studies included.

The results varied from high to very low certainty of the evidence, according to the GRADE classification. We found several types of systematic errors in the studies: random sequence generation (selection bias, in 4 studies), incomplete outcome data (attrition bias, in 3 studies), and imprecision (observed within all the studies, in 303 outcomes evaluated representing 83% of the total).

We found for the study with the lowest risk of bias ( Irene *et al.*, 2019) a range for the certainty of the evidence between high (for fertility-related concerns scale scores ≤3 and improvement with no or low fertility or pregnancy concerns) to moderate certainty of the evidence (for improvement in at least one women’s health issue and 50% decrease in the hot flash score), as described in Table 3a.

**Table 3a.** Summary of findings: Low risk of bias ( Irene *et al.*, 2019).

Patient or population: young breast cancer survivors Setting: web-based, women’s health survivorship care plan (SCP) Intervention: implementation of the recommendations of the CPG Comparison: usual care Outcomes: improve hot flashes, fertility-related concerns, contraception, and vaginal symptoms						
Outcomes	Effects		Relative effect (95% CI)	№ of participants (studies)	Certainty of the evidence (GRADE)	Importance
	With usual care	With web-based women’s health survivorship care plan (SCP)				
Fertility-related concerns scale scores ≤3; assessed with: Reproductive Concerns After Cancer scale (RCAC) follow-up: mean 24 weeks	146 per 1,000	279 per 1,000 (158 to 452)	OR 2.27 (1.10 to 4.84)	182 (1 RCT)	⨁⨁⨁⨁ High ^a^ ^,^ ^b^ ^,^ ^h^	Important
Improvement with no or low fertility or pregnancy concerns; assessed with: Reproductive Concerns After Cancer scale (RCAC), follow-up: mean 24 weeks	304 per 1,000	533 per 1,000 (329 to 733)	OR 2.61 (1.12 to 6.29)	91 (1 RCT)	⨁⨁⨁⨁ High ^a^ ^,^ ^c^ ^,^ ^h^	Important
Improvement in at least one women’s health issue, follow-up: mean 24 weeks	573 per 1,000	709 per 1,000 (570 to 820)	OR 1.82 (0.99 to 3.40)	182 (1 RCT)	⨁⨁⨁◯ Moderate ^a^ ^,^ ^e^	Important
50% decrease in hot flash score; assessed with: Hot Flashes Score, follow-up: mean 24 weeks	552 per 1,000	578 per 1,000 (413 to 729)	OR 1.11 (0.57 to 2.18)	182 (1 RCT)	⨁⨁⨁◯ Moderate ^a^ ^,^ ^f^	Important

CI: confidence interval; OR: odds ratio.

**GRADE Working Group grades of evidence:** High certainty: we are very confident that the true effect lies close to that of the estimate of the effect. Moderate certainty: we are moderately confident in the effect estimate: the true effect is likely to be close to the estimate of the effect, but there is a possibility that it is substantially different. Low certainty: our confidence in the effect estimate is limited: the true effect may be substantially different from the estimate of the effect. Very low certainty: we have very little confidence in the effect estimate: the true effect is likely to be substantially different from the estimate of effect.

^a^ Risk of bias. Lack of blinding in clinical staff.

^b^ Imprecision. 95% CI: 1.10 to 4.84. p: 0.03. Statistically significant.

^c^ Imprecision. 95% CI: 1.12 to 6.29. p: 0.03. Statistically significant.

^e^ Imprecision. 95% CI: 0.99 to 3.40. p: 0.057. Not statistically significant. Downgraded -1 for imprecision.

^f^ Imprecision. 95% CI: 0.57 to 2.18. p: 0.75. Not statistically significant. Downgraded -1 for imprecision.

^h^ Strong association. OR > 2. Large effect. Upgraded +1.

In the case of Park *et al.* (2019) study reporting a high risk of bias, we found a range for the certainty of the evidence between low (moderate MET-min/wk and walk MET-min/wk at six months) and very low certainty of the evidence (Moderate MET-min/wk and Walk MET-min/wk at 12 months), as described in Table 3b. Our results after grading the certainty of the evidence for both studies were consistent with the findings from the RoB2 instrument.

**Table 3b.** Summary of findings: High risk of bias ( Park *et al.*, 2019).

Patient or population: peri-menopausal and early postmenopausal female cancer survival Setting: Yale Fitness Intervention Trial Intervention: adherence to the ACS guidelines Comparison: usual care Outcome: improvement of the quality of life (QoL)						
Outcomes	Mean effects min/wk ± SD		Absolute effect (95% CI)	№ of participants (studies)	Certainty of the evidence (GRADE)	Importance
	With usual care	With adherence to the ACS guidelines				
Moderate MET-min/wk; assessed with: International Physical Activity Questionnaire (IPAQ), follow-up: mean 6 months	33.15 ± 123.87	681.23 ± 127.38	648.08 min/wk (606.091 to 690.069)	138 (1 RCT)	⨁⨁◯◯ Low ^a^ ^,^ ^b^	Important
Moderate MET-min/wk; assessed with: International Physical Activity Questionnaire (IPAQ), follow-up: mean 12 months	38.19 ± 128.25	115.69 ± 133.59	77.5 min/wk (32.289 to 122.711)	130 (1 RCT)	⨁◯◯◯ Very low ^a^ ^,^ ^c^	Important
Walk MET-min/wk; assessed with: International Physical Activity Questionnaire (IPAQ), follow-up: mean 6 months	-178.80 ± 124.52	236.63 ± 127.78	415.43 min/wk (373.268 to 457.592)	138 (1 RCT)	⨁⨁◯◯ Low ^a^ ^,^ ^d^	Important
Walk MET-min/wk; assessed with: International Physical Activity Questionnaire (IPAQ), follow-up: mean 12 months	-126.27 ± 127.75	157.37 ± 132.40	283.64 min/wk (238.718 to 328.562)	130 (1 RCT)	⨁◯◯◯ Very low ^a^ ^,^ ^e^	Important

CI: confidence interval.

**GRADE Working Group grades of evidence:** High certainty: we are very confident that the true effect lies close to that of the estimate of the effect. Moderate certainty: we are moderately confident in the effect estimate: the true effect is likely to be close to the estimate of the effect, but there is a possibility that it is substantially different. Low certainty: our confidence in the effect estimate is limited: the true effect may be substantially different from the estimate of the effect. Very low certainty: we have very little confidence in the effect estimate: the true effect is likely to be substantially different from the estimate of effect.

^a^ Risk of bias. Lack of allocation concealment. Selection bias. Lack of blinding. Downgraded -2 for risk of bias.

^b^ Imprecision. 95% CI: 606.091 to 690.069. p: 0.0004. Statistically significant.

^c^ Imprecision. 95% CI: 32.289 to 122.711. p: 0.67. Not statistically significant. Downgraded -1 for imprecision.

^d^ Imprecision. 95% CI: 373.268 to 457.592. p: 0.02. Statistically significant.

^e^ Imprecision. 95% CI: 238.718 to 328.562. p: 0.12. Not statistically significant. Downgraded -1 for imprecision.

### Assessment of the studies outcomes

The outcomes were grouped in simple relative and absolute numbers. A global estimate of the measurements of the effects included in the studies is lacking because of the significant variability of measuring units combined with the clinical heterogeneity found among the studies included.

There was significant variability in the measurement of the outcomes reported in the studies. Most were continuous, e.g., to assess the quality of life, which corresponds to the dimension of patient outcome. A total of 362 were included in the 11 RCTs evaluated; 11 (3%) corresponded to the health care structure dimension, 51 (14%) interventions to the dimension process and 302 (83%) interventions to the dimension of patient outcomes. A broader description of the main findings by the dimensions evaluated in the included studies is available as extended data, Appendix 7 ( Ramírez-Morera *et al.*, 2022).

Regarding the impact of using EB-CPG, we found 303 (83%) interventions with no significant difference between the control and experimental groups. The outcome favoured the control group in 4 (1%). Three outcomes interfered with the patient’s adherence (predictable variables: age, marital status, and hot flashes), as reported by Maly *et al.* (2017). Also, the fourth outcome informed by Klinkhammer-Schalke *et al.* (2012) reported rates of therapeutic options for Physiotherapy 16 (experimental group) vs 30 (control group), p: <0.02, at six months. The result favoured the intervention group for 57 interventions (16%) ( Table 4).

**Table 4.** Summary of results for all included studies by dimensions and effect (n=11).

	Citation	Intervention						
		Dimension			In favour CPG n (%)	Equal n (%)	In favour control n (%)	Total n (%)
		Structure	Process	Patient outcome				
1	Boekhout *et al.*, 2015	7 (17%)	20 (49%)	14 (34%)	8 (17%)	33 (83%)	0	41 (100%)
2	Greenlee *et al.*, 2016	0	0	16 (100%)	4 (25%)	12 (75%)	0	16 (100%)
3	Grunfeld *et al.*, 2011	0	6 (25%)	18 (75%)	3 (13%)	21 (87%)	0	24 (100%)
4	Hershman *et al.*, 2013	0	0	27 (100%)	2 (8%)	25 (92%)	0	27 (100%)
5	Irene *et al.*, 2019	0	5 (42%)	7 (58%)	3 (25%)	9 (75%)	0	12 (100%)
6	Klinkhammer-Schalke *et al.*, 2012	0	8 (12%)	60 (88%)	10 (15%)	57 (84%)	1 (1%)	68 (100%)
7	Knobf *et al.*, 2016	0	0	24 (100%)	7 (29%)	17 (71%)	0	24 (100%)
8	Maly *et al.*, 2017	2 (5%)	10 (23%)	32 (72%)	3 (7%)	38 (86%)	3 (7%)	44 (100%)
9	Park *et al.*, 2015	2 (5%)	0	38 (95%)	6 (15%)	34 (85%)	0	40 (100%)
10	Park *et al.*, 2019	0	0	53 (100%)	6 (11%)	47 (89%)	0	53 (100%)
11	Smith-Turchyn *et al.*, 2020	0	0	13 (100%)	3 (23%)	10 (77%)	0	13 (100%)
TOTAL		11 (3%)	51 (14%)	302 (83%)	57 (16%)	303 (83%)	4 (1%)	364 (100%)

## Discussion

For more than two decades, governmental and non-governmental institutions have been making economic and methodological efforts to develop more and better quality EB-CPGs, seeking to deal with different issues most healthcare systems face. Such as the ageing population, rising costs motivated by increased demand for quality care, increasingly expensive emerging health technologies, variability in the provision of health by presuming that part of this disparity could cause inadequate care (either overuse or underuse of supplies), and the desire for clinicians and patients to provide and to receive, respectively, the best possible care with measurable clinical effect. However, it still appears that some of these EB-CPGs are far from contributing to an effective, standardised clinical practice based on the best available evidence ( Woolf *et al.*, 1999; IOM, 2011; Alonso-Coello *et al.*, 2010). We agree with Woolf *et al.* (1999) that EB-CPGs that promote proven benefits and discourage ineffective interventions could reduce morbidity and mortality, and improve quality of life, at least for some conditions. EB-CPGs can also improve the consistency of care.

The effects of the recommendations in the interventions included in the 11 RCTs considered in this project were in the structure of medical care (3%) and the care provided (14%); both were the least explored. Surprisingly, patient outcomes were the most evaluated domain (83%), with significant results in 43 of 302 (14%), representing 75% of all significant results.

This fact could lead us to suppose that researchers finally focused on the importance of evaluating the patient health, solely prioritising evaluating the adherence to the CPG (assess the process dimension). They are trying to glimpse more clearly what the use of the CPG represents for patients and not only for clinical staff. As this review considered breast cancer, we could not ignore that there is more social and economic pressure to know the patient outcome.

Grimshaw and Russell (1993) findings described in their systematic review, “Effect of clinical guidelines on medical practice: a systematic review of rigorous evaluations”, reported more than 80% significant improvements among the included studies. Contrasting their results, in this second systematic review, we found 57 interventions on breast cancer in favour of the use of EB-CPGs distributed in all the studies included (16% of all the interventions evaluated). Then, compared to our first systematic review, only half of the measures with statistically significant results favour using EB-CPGs ( Ramírez-Morera *et al.*, 2019). However, as 75% of these results were found for the dimension of results in patients, we keep optimistic about the finding on incoming reviews an increasingly more significant impact.

Four studies (36%) reported a high risk of bias ( Knobf *et al.*, 2016; Klinkhammer-Schalke *et al.*, 2012; Maly *et al.*, 2017; Park *et al.*, 2019), and some concerns arise from 6 (55%) studies ( Grunfeld *et al.*, 2011; Hershman *et al.*, 2013; Boekhout *et al.*, 2015; Park *et al.*, 2015; Greenlee *et al.*, 2016; Smith-Turchyn *et al.*, 2020). Because of that, we agree with Ivers *et al.* (2012) that EB-CPGs must be evaluated, including more remarkable methodological quality studies to provide feedback and corrective measures for clinical practice through audits, promoting improvements in the quality of care.

We also concur with Lugtenberg *et al.* (2009) about the need to focus on the strength of the recommendations for determining what factors influence the use of the guidelines and the improvement of the results of the patients.

Then we identified the need to perform recommendations distinguishing between the level of certainty of evidence (stratified analysis) as this could have a more significant impact on the results when the best available certainty of evidence recommendation is implemented. We keep in mind that explicit EB-CPG improves clinical practice when introduced within a context of rigorous evaluations ( Grimshaw & Russell, 1993; Ricci-Cabello *et al.*, 2020).

Some studies included in the review five reported outcomes in the process area ( Boekhout *et al.*, 2015; Grunfeld *et al.*, 2011; Irene *et al.*, 2019; Klinkhammer-Schalke *et al.*, 2012; Maly *et al.*, 2017). All of them described favourable results for the intervention in 14 of 51 measurements (27%). This fact reflects that researchers have continued to endeavour to measure when EB-CPGs should be used or not, but this time to a much lesser extent (14% vs 64%) when compared to the first review.

Lugtenberg *et al.* (2009) reported that the size of the effects observed in their systematic review varied considerably between the recommendations within the guidelines. We repeated this finding in our study and the previous one ( Ramírez-Morera *et al.*, 2019). We found that in many evaluated interventions (303, 83%), the use of EB-CPG did not reflect any impact in any dimension. The approach followed to report the current effectiveness of EB-CPGs remains incomplete ( Woolf *et al.*, 1999), and a strategy that captures better results has not yet been found.

EB-CPGs may contribute to improving the quality of healthcare. However, it is still necessary to integrate them with strategies that enhance their use and effect, such as academic and educational visits as part of ongoing training programs ( O’Brien *et al.*, 2007). Repeatedly, advocates for EB-CPGs consider their only existence as a magic solution to solve health care problems; however, they ignore other practical actions that should be implemented along with the guidelines ( Woolf *et al.*, 1999).

EB-CPG has an essential role when clinicians do not clearly know the appropriate practice and which scientific evidence should support their decisions ( Woolf *et al.*, 1999). Then, EB-CPG developers should be vigilant in identifying these needs to help close this information gap and increase the enthusiasm for employing them.

## Conclusions

Developing strategies for a more standardised implementation of the EB-CPGs through structured programs within health systems is essential. Improving the awareness of clinical staff about the possibility of enhancing clinical practice and patient outcomes by using evidence-based recommendations with an expected effect is a need.

There is an imbalance between the number of EB-CPGs developed for breast cancer and the number of high-quality studies evaluating their effectiveness. Due to the limited results found on the benefit of using EB-CPG, we must continue investigating the subject. We could gradually structure a more robust hypothesis about the variables influencing this issue.

The variation in the effects found for the recommendations included in the EB-CPGs suggests that it would be helpful to change strategies and focus on the analysis of the limitations of adherence and on designing implementation approaches adapting each recommendation.

In addition, future RCTs should distinguish the levels of certainty of the evidence supporting each recommendation in their evaluations. Researchers should focus on evaluating recommendations expected to have the most significant impact (superior levels of certainty of the evidence: High or Moderate).

More research is necessary to define which factors related to the implementation of EB-CPG and its specific recommendations are essential to predict the application of EB-CPG and, therefore, achieve better patient results.

### Implications for the practice

This systematic review aimed to support the development of programs evaluating the effects of EB-CPG on the quality of health care. Also, to provide reliable evidence sustaining the decision-making process related to the production of EB-CPGs.

Even when some of the results of this systematic review were statistically significant, supporting the use of EB-CPG as a tool to improve clinical practice and quality of care, the results of this review need to be interpreted with caution.

The implementation of EB-CPGs must consider the differences in the measures of effect to define customised approaches and specific recommendations within the guideline to enhance health care.

For the adequate implementation of EB-CPGs, it is necessary to consider the possible costs, risks, and benefits and the expected effects derived from EB-CPGs recommendations. The efforts to build EB-CPGs must be complemented with psychosocial strategies promoting health personnel to follow the recommendations of the EB-CPG and evaluate their impact.

Greater methodological rigour in the development of CPGs is needed. It is also required to carry out this process within a standardised formal program in the health systems. Greater credibility could be achieved if recommendations are based on the best available evidence, improving the credibility of their positive effect among health personnel. It could also lead to a more willingness to implement the CPGs and to participate in evaluating their impact.

### Implications for the research

This study corresponds to the second systematic review of a series of studies aiming to assess the effect of Evidence-Based Clinical Practice Guidelines (EB-CPGs) on improving health care quality. Our next and last project will investigate Covid-19 disease.

Due to recent research in this field, and the results of this study were not conclusive, more research is necessary to evaluate how EB-CPGs could impact the quality of health care, especially emphasising fewer investigated areas, such as the structure of healthcare services and patient outcomes.

Additionally, the design and methodological rigour applied to future RCTs should improve by considering the certainty of the evidence supporting the EB-CPGs recommendations. Besides, focusing on those with a greater level of evidence (high or moderate) which could lead to determining more clearly the effect that these recommendations have on the quality of health care.

## Data (and software) availability

### Data availability

*Underlying data*

All data underlying the results are available as part of the article, and no additional source data are required.

*Extended data*

Open Science Framework: Extended data for the second SR CPG Breast Cancer. DOI: https://osf.io/6h9pm/?view_only= ( Ramírez-Morera *et al.*, 2022).

This project contains the following extended data:

Appendix 1. Advanced search strategy and results.pdf

Appendix 2. List of excluded studies and reasons for exclusion.pdf

Appendix 3. List of selected studies after screening and assessing the full text.pdf

Appendix 4. Characteristics of the included studies.pdf

Appendix 5. List of the CPGs examined in the included studies.pdf

Appendix 6. Risk of Bias 2 assessment.pdf

Appendix 7. Main findings by dimensions of the included studies.pdf

*Reporting guidelines*

We followed the PRISMA 2020 statement for reporting systematic reviews ( Page *et al.*, 2021). We did the PRISMA 2020 checklist and the flow diagram for new systematic reviews, which included searches of databases, registers, and other sources.

Open Science Framework: PRISMA checklist and flow chart for Effects of evidence-based clinical practice guidelines for breast cancer in health care quality improvements. A second systematic review. DOI: https://osf.io/k7f5x/?view_only=7d6b4a63853c43e4b233424ea226e66a.

## Author contributions

**Anggie Ramírez-Morera:** Conceptualisation, Data Curation, Formal Analysis, Funding Acquisition, Methodology, Project Administration, Writing – Original Draft Preparation, Writing – Review & Editing.

**Mario Tristán:** Conceptualisation, Data Curation, Formal Analysis, Funding Acquisition, Methodology, Resources, Software, Supervision, Writing – Original Draft Preparation, Writing – Review & Editing.

**Jordan Salazar-Vargas:** Data Curation, Formal Analysis, Funding Acquisition, Resources, Writing – Original Draft Preparation, Writing – Review & Editing.

**Ana Leonor Rivera-Chavarría:** Data Curation, Writing – Original Draft Preparation, Writing – Review & Editing.

## Leading author information

Anggie Ramírez-Morera is PhD candidate Program in Biomedical Research Methodology and Public Health, Universitat Autònoma de Barcelona.
