# Supplementary material for: Effects of evidence-based clinical practice guidelines for breast cancer in health care quality improvements. A second systematic review
Source: F1000Res. 2022 Dec 5;11:1213. Originally published 2022 Oct 25. [Version 2] doi: 10.12688/f1000research.126126.2 (PMC9780606; doi:10.12688/f1000research.126126.2)
Supplement: Peer review history

# Peer review: Reviewer response for version 2

**DOI:** 10.5256/f1000research.141742.r157384

**Published:** 2022-12-22

**Authors:** Ignacio Marín León

**Affiliations:** 1 Internal Medicine, Virgen del Rocío University Hospital, Seville, Spain

I agree with the current version of the article

Are the rationale for, and objectives of, the Systematic Review clearly stated?

Yes

Is the statistical analysis and its interpretation appropriate?

Yes

Are sufficient details of the methods and analysis provided to allow replication by others?

Partly

Are the conclusions drawn adequately supported by the results presented in the review?

Yes

Reviewer Expertise:

Clinical Epidemiology

I confirm that I have read this submission and believe that I have an appropriate level of expertise to confirm that it is of an acceptable scientific standard.

# Peer review: Reviewer response for version 2

**DOI:** 10.5256/f1000research.141742.r158163

**Published:** 2022-12-14

**Authors:** Wanderley Marques Bernardo

**Affiliations:** 1 Evidence Based Medicine Department, University of Sao Paulo, Sao Paulo, Brazil; 2 Brazilian Medical Association Guidelines Coordinator, Brazilian Medical Association, Sao Paulo, Brazil

This is a systematic review of evidence-based guidelines related to breast cancer, and the impact of their use in clinical practice. The method used by the authors achieved all quality items of a systematic review. The authors could not do a meta **-**analysis due to the heterogeneity of the 11 RCTs selected, and the 364 interventions involved. The main limitation of the systematic review is the expression of results that was mainly descriptive, in spite of some attempt at a quantitative reference to differences between control and experimental groups. But these differences were not associated with specific outcomes. The discussion and conclusions tell us about these limitations and reinforce the importance of considering and focusing on the 3 dimensions when EB guidelines are constructed. This systematic review brings a good model to be reproduced in other clinical subjects of guidelines, considering the adherence and the clinical impacts.

 The first quality item of this study is the objective of not only critically evaluating EB guidelines, but mainly evaluating their usefulness measured by clinical outcomes in their implementation.

 Analyzing patients with breast cancer as the focus of management, while bringing relevant interventions and outcomes, also brings great variability between conducts, increasing the heterogeneity of the analyses.

 In the method, the critical use of three domains (structure, process and result) increases the ability to assess the implementation of the guidelines on the subject.

 The search method, selection (eligibility criteria), data extraction, risk of bias assessment, and quality of evidence are fully reproducible and adequate.

 There is a limitation in the expression of results due to the already expected heterogeneity between the randomized clinical trials included in the guidelines implementation environment (11 RCTs and more than 300 interventions involved).

 The analysis, fundamentally supported by the “outcomes” domain, discourages the use of guidelines, since, in the majority, the results are similar when using or not using a standardized conduct. However, this analysis is not specific about the interventions adopted and measured outcomes, which in addition to not allowing a quantitative expression, does not allow for analyzing the reasons for the absence of differences in the implementation of the recommendations.

 And the questions that remain are: is there still any doubt whether the homogenization of medical practice produces benefits and reduces harm to patients? Is this question still open to investigation?

Are the rationale for, and objectives of, the Systematic Review clearly stated?

Yes

Is the statistical analysis and its interpretation appropriate?

Not applicable

Are sufficient details of the methods and analysis provided to allow replication by others?

Yes

Are the conclusions drawn adequately supported by the results presented in the review?

Yes

Reviewer Expertise:

Evidence Based Medicine, Systematic Review and Guidelines

I confirm that I have read this submission and believe that I have an appropriate level of expertise to confirm that it is of an acceptable scientific standard.

# Peer review: Reviewer response for version 1

**DOI:** 10.5256/f1000research.138504.r154242

**Published:** 2022-11-04

**Authors:** Ignacio Marín León

**Affiliations:** 1 Internal Medicine, Virgen del Rocío University Hospital, Seville, Spain

This is an original paper of substantial importance addressing CPG implementation effect. Mostly a very sensible, necessary and well-developed document. In general, the writing is clear and concise, however, needs some editing to avoid unnecessary repetition (i.e. the three domains of Donabedian proposal, and some others). The effect of the implementation of 11 guidelines identified after a rigorous and well-done literature search is presented, as detailed in Methods and Figure 1.

 The reviewer has some concerns and suggestions regarding Methods (GRADE use) and Discussion that need to be addressed to improve the manuscript.

 An article that is worthy of being indexed.

**Major Comments**

**Methods:**

*Data extraction, page 4, last paragraph, “An analysis of the quality of the evidence…we assessed the certainty...”*: It is unclear how this was done. GRADE is a very labour-intensive and specific methodology, and to perform a GRADE evaluation, the authors have two options: a) simply seek the strength of evidence from the original implemented guideline included in the review, or b) evaluate and critically assess original articles cited in the guidelines reviewed, when they did not use GRADE methodology. This needs clarification on what the authors mean. In addition, readers do not need the authors to explain what GRADE method is.

**Results:**

*Quality of evidence assessment, page 7*: This section should be clarified in accordance with previous comments on the use of GRADE.

**Discussion:**

1. The structure of this section does not agree with the established "norms" on how the "discussion" section should be drafted. The main findings of the study should first be highlighted in relation to the objective of the study, and in a second step explain the causes of such findings and contrast with similar studies in the available literature. Thus, the large first paragraph could be moved down (and shortened) in the text.
2. The reviewer does not agree with the statements made in the current third paragraph, in the sense of contrasting the effect on the "process of care" with the effect on "patient outcomes". There are RCTs that relate the implementation of guidelines process of care with the appropriateness and necessity of care for patients' benefits (i.e. see Romero *et al. *(2005) ^1^).
3. Some comment on study weaknesses is missing.

**Minor Comments**:

**Abstract:**

 It is required that the clinical problem addressed in the study (breast cancer) appears in the abstract and keywords.

**Introduction:**

*In the sixth paragraph*: *“Although survival has improved…..”*: There is a huge debate whether the improved survival depends on the early diagnosis or the improvement of knowledge of the natural history and new treatments of breast cancer (Gøtzsche *et al. *(2013) ^2^, Elmore *et al. *(2005) ^3^). Thus, some sentences should be complemented with “...of early detection programs and treatment improvement”.

**Discussion:**

 The comments on the usefulness of EB-CPG could be shortened.

Are the rationale for, and objectives of, the Systematic Review clearly stated?

Yes

Is the statistical analysis and its interpretation appropriate?

Yes

Are sufficient details of the methods and analysis provided to allow replication by others?

Partly

Are the conclusions drawn adequately supported by the results presented in the review?

Yes

Reviewer Expertise:

Clinical Epidemiology

I confirm that I have read this submission and believe that I have an appropriate level of expertise to confirm that it is of an acceptable scientific standard, however I have significant reservations, as outlined above.

## References

[1] : Effectiveness of a Multifactorial Strategy for Implementing Clinical Guidelines on Unstable Angina: Cluster Randomized Trial. *Revista Española de Cardiología (English Edition)* .2005;58(6) : 10.1016/S1885-5857(06)60250-8 640-648 10.1016/S1885-5857(06)60250-8 15970119

[2] : Screening for breast cancer with mammography. *Cochrane Database Syst Rev* .2013; 10.1002/14651858.CD001877.pub5 CD001877 10.1002/14651858.CD001877.pub5 23737396PMC6464778

[3] : Screening for breast cancer. *JAMA* .2005;293(10) : 10.1001/jama.293.10.1245 1245-56 10.1001/jama.293.10.1245 15755947PMC3149836

# Response

**Published:** 2022-11-30

**Authors:** Anggie Ramirez

Dear Dr. Ignacio Marin,

 We would like to thank you for agreeing to participate in the review of our research. We consider that your contributions are very valuable to improve our work.

 Regarding your comments, we describe as follows the research team's position on each of them:

**A. Concerning the analysis of the certainty of the evidence, you wrote:**

***Methods:***

*Data extraction, page 4, last paragraph, "An analysis of the quality of the evidence…we assessed the certainty...": It is unclear how this was done. GRADE is a very labour-intensive and specific methodology, and to perform a GRADE evaluation, the authors have two options: a) simply seek the strength of evidence from the original implemented guideline included in the review, or b) evaluate and critically assess original articles cited in the guidelines reviewed, when they did not use GRADE methodology. This needs clarification on what the authors mean. In addition, readers do not need the authors to explain what GRADE method is.*

***Results:***

*Quality of evidence assessment, page 7: This section should be clarified in accordance with previous comments on the use of GRADE.*

**Answer to #A:**

 We agree with you about the options using the GRADE method to develop or adapt a Clinical Practice Guideline. However, we used the GRADE method to assess the certainty of the evidence only for the RCTs that met the inclusion criteria. Therefore, it does not comprise part of the objective and scope of this systematic review to evaluate the evidence included in the CPGs that these studies reviewed.

 We wrote as part of the Methods section:

 The studies showing the lowest risk of bias (Irene et al., 2019) and one resulting in the highest risk of bias (Park et al., 2019) were chosen to be evaluated with the GRADE methodology and to build a summary of the findings. We decided to grade only four outcomes per study described, including two reporting statistically significant results and two not statistically significant results. Performing a GRADE table provided the rank of possible grades for the certainty of the evidence found in the 362 interventions from the 11 studies included.

 Hence, we do not consider it necessary to modify the text in the section "Quality of evidence assessment". Then, we want to clarify that it is part of the objective and scope of this systematic review to evaluate the certainty of the evidence of the RCTs included and not of the EB-CPGs that they evaluated.

**B. About the article's Discussion section, you wrote:**

**B.1**

*The structure of this section does not agree with the established "norms" on how the "discussion" section should be drafted. The main findings of the study should first be highlighted in relation to the objective of the study, and in a second step explain the causes of such findings and contrast with similar studies in the available literature. Thus, the large first paragraph could be moved down (and shortened) in the text.*

**Answer to #B.1:**

 It is relevant to clarify that our article complies with F1000Research guidelines for preparing a Systematic Review article. The journal requests the main body section as follows:

 Main Body: The format of the main body of the article is flexible: it should be concise, making it easy to read and review, and presented in a format that is appropriate for the type of study presented. For most Systematic Reviews, the following standard format will be the most appropriate: Introduction, Methods, Results, Conclusions/Discussion.

 Also, we consider that the aim of the discussion was achieved because we highlighted our findings and contrasted them with similar research. We must add that we have also complied with the PRISMA 2020 statement checklist (Page et al., 2021[1]). About the discussion, our study meets with the following items:

 23a Provide a general interpretation of the results in the context of other evidence.

 23b Discuss any limitations of the evidence included in the review.

 23c Discuss any limitations of the review processes used.

 23d Discuss the implications of the results for practice, policy, and future research.

 Therefore, considering that neither of the indications describes an expected order of ideas as you have indicated, the authors alleged it convenient to include this first paragraph to provide a context about the world situation regarding the efforts to develop guidelines and the expectations placed by health caregivers on their effect. Also, to link this finding with the findings in our study.

 Consequently, as the first paragraph of this section is congruent with the aim of this systematic review, we consider it appropriate to keep the paragraph unchanged.

**B.2 Additionally, regarding your comment about the third paragraph of the discussion section:**

*The reviewer does not agree with the statements made in the current third paragraph, in the sense of contrasting the effect on the "process of care" with the effect on "patient outcomes". There are RCTs that relate the implementation of guidelines process of care with the appropriateness and necessity of care for patients' benefits (i.e. see Romero et al. (2005)[2])*.

 We would like to explain the following:

**Answer #B.2:**

 This statement refers to the findings we got from the first systematic review (Ramírez-Morera et al., 2019[3]) that focused on the impact of CPGs in the cardiovascular approach; at that time, we included nine RCTs. Therefore, we decided to compare the results obtained in this second SR to our previous research regarding the number of interventions evaluated according to the dimensions described by Donabedian (1988)[4].

 This report corresponds to the result of a Systematic Review that includes only Randomized Controlled Clinical Trials (RCTs) whose objective was to measure the impact that Clinical Practice Guidelines have produced on the quality of health care. This review's authors analyzed the RCT measurements' results according to the framework proposed by Avedis Donabedian ^^[5]^^ (7 January 1919 – 9 November 2000). Therefore, what has been understood by the implementation of clinical practice guidelines, as defined in the UK glossary published by NICE, adheres to the definition mentioned earlier: "implementation, The process of putting guideline recommendations into practice" (NICE 2014) ^^[6]^^. Evaluation components of CPG use, such as clinical auditing, can be of great value. However, clinical auditing has not been disseminated to be available in other countries, predominantly low-income countries.

 In 2008, Dutch authors Lugtenberg M, Burgers JS and Westert GP published an excellent systematic review ^^[7]^^ using the quality of care model by Donabedian. This publication has been very inspiring for the authors of this series of systematic reviews. This review mainly includes RCT studies and some before and after studies done on CPG made in the Netherlands, a country with a long tradition in the design and use of CPG. Some of their results are encouraging, although other aspects were weak, such as the impact on patient's health, which should be considered a very relevant result.

 Due to the nature of the data, it was impossible to have a quantitative synthesis, only qualitative. But it did not inhibit us from assessing the certainty of the evidence for the results of the included studies after searching and screening according to the inclusion criteria.

 To determine the confidence of the evidence, we have followed Cochrane's recommendations using the tools developed by GRADE. GRADE is a tool that requires knowledge of the essential elements of effective measurement and its potential biases. The authors of this SR are trained to complete this task with adequate competency.

**B.3** **You also wrote: *Some comment on study weaknesses is missing.***

**Answer #B.3:**

 We did not write within a specific section of our article what the research's weaknesses are. The weaknesses of the study are described throughout different statements, for example, when describing the risk of bias assessment for the included studies. We also identified many EB-CPGs developed in contrast to the few numbers evaluated through formal RCT-type investigations.

 We also wrote about the implications for research:

 Due to recent research in this field, and the results of this study were not conclusive, more research is necessary to evaluate how EB-CPGs could impact the quality of health care, especially emphasising fewer investigated areas, such as the structure of healthcare services and patient outcomes.

 Additionally, the design and methodological rigour applied to future RCTs should improve by considering the certainty of the evidence supporting the EB-CPGs recommendations. Besides, focusing on those with a greater level of evidence (high or moderate) which could lead to determining more clearly the effect that these recommendations have on the quality of health care.

**C. About your minor comments** :

**C.1** **First, about the abstract section you wrote:**

***Abstract: **It is required that the clinical problem addressed in the study (breast cancer) appears in the abstract and keywords.*

**Answer #C.1:**

 We agree to add "breast cancer" as a keyword.

**C.2** **Second, concerning the introduction section, you wrote:**

**Introduction:**

*In the sixth paragraph: "Although survival has improved…..": There is a huge debate whether the improved survival depends on the early diagnosis or the improvement of knowledge of the natural history and new treatments of breast cancer (Gøtzsche et al. (2013)[8], Elmore et al. (2005)[9]). Thus, some sentences should be complemented with "...of early detection programs and treatment improvement".*

**Answer #C.2:**

 We agree to add the suggested text.

**C.3** **Third, referring to the discussion section, you wrote:**

*The comments on the usefulness of EB-CPG could be shortened.*

**Answer #C.3:**

 The authors alleged it is convenient to describe the usefulness of EB-CPG because it is congruent with the aim of this systematic review. Therefore, we disagree with the modification suggested.

**C.4** **Fourth and last, you wrote that there are 'partly' details of the methods and analysis provided to allow replication by others.**

**Answer #C.4:**

 Full details on methods and further analyses to allow replication by others can also be accessed through the research protocol, registered at PROSPERO: CRD42020205594.

**References**

 [1] Page et al. (2021). The PRISMA 2020 statement: an updated guideline for reporting systematic reviews. BMJ (Clinical Research Ed.), 372, p.n71. [Online]. Available at: doi:10.1136/bmj.n71.

 [2] Romero A, Alonso C, Marín I, Grimshaw J, et al.: Effectiveness of a Multifactorial Strategy for Implementing Clinical Guidelines on Unstable Angina: Cluster Randomized Trial. *Revista Española de Cardiología (English Edition)*. 2005; 58 (6): 640-648 

 [3] Ramírez-Morera, A., Tristan, M. and Vazquez, J. C. (2019). Effects of evidence-based clinical practice guidelines in cardiovascular health care quality improvements: A systematic review [version 3; peer review: 2 approved]. F1000Research 2019, 8:1041[Online]. Available at: https://doi.org/10.12688/f1000research.18865.3

 [4] Donabedian, A. (1988). The quality of care. How can it be assessed? The Journal of the American Medical Association, 260 (12), pp.1743–1748. [Online]. Available at: doi:10.1001/jama.260.12.1743.

 [5] Donabedian model. (2022, August 8). In *Wikipedia*. https://en.wikipedia.org/wiki/Donabedian_model

 [6] Glossary | Developing NICE guidelines: the manual | Guidance | NICE [WWW Document], 2014 LAST UPDATED 18 January 2022. URL https://www.nice.org.uk/process/pmg20/chapter/glossary

 [7] Lugtenberg, M., Burgers, J.S., Westert, G.P., 2009. Effects of evidence-based clinical practice guidelines on quality of care: a systematic review. Qual. Saf. Health Care 18, 385–392. doi:10.1136/qshc.2008.028043

 [8] Gøtzsche PC, Jørgensen KJ: Screening for breast cancer with mammography. *Cochrane Database Syst Rev*. 2013. CD001877 

 [9] Elmore JG, Armstrong K, Lehman CD, Fletcher SW: Screening for breast cancer. *JAMA*. 2005; 293 (10): 1245-56
